# Recent Information on Vitamin D Deficiency in an Adult Korean Population Visiting Local Clinics and Hospitals

**DOI:** 10.3390/nu14091978

**Published:** 2022-05-09

**Authors:** Rihwa Choi, Sung-Eun Cho, Sang Gon Lee, Eun Hee Lee

**Affiliations:** 1 Department of Laboratory Medicine, Green Cross Laboratories, Yongin 16924, Korea; pirate0720@naver.com (R.C.); secho1206!!@gclabs.co.kr (S.-E.C.); 2 Department of Laboratory Medicine and Genetics, Samsung Medical Center, Sungkyunkwan University School of Medicine, Seoul 06351, Korea; 3 Green Cross Laboratories, Yongin 16924, Korea

**Keywords:** vitamin D, 25-hydroxy vitamin D, deficiency, Korea

## Abstract

We retrospectively reviewed serum 25-hydroxy vitamin D (25(OH)D) test results from an adult Korean population visiting local clinics and hospitals between July 2017 and December 2021 to gather recent information on the prevalence of vitamin D deficiency. The prevalence of vitamin D deficiency status was investigated according to criteria offered by various clinical guidelines. During the study period, 180,289 subjects (29,658 men and 150,631 women) were tested for 25(OH)D. The overall prevalence rates of vitamin D deficiency status based on 25(OH)D level were as follows: 0.4% for <5 ng/mL, 12.5% for <10 ng/mL, 20.6% for <12 ng/mL, 49.4% for <20 ng/mL, and <75.3% for <30 ng/mL. Women tested their 25(OH)D level more frequently than men, and the overall prevalence of 25(OH)D < 10 ng/mL was higher among women than men, while that of 25(OH)D <30 ng/mL was lower among women than men. Among age groups, the prevalence of 25(OH)D <30 ng/mL was higher in younger patients (20s–40s, 79.6–85.5%) than older ones (≥50 years, 62.6–69.2%). The overall prevalence of vitamin D deficiency decreased over time from 2018 to 2021. Future studies are needed to clarify the clinical impact of this change.

## 1. Introduction

Vitamin D, a fat-soluble secosteroid, is essential for calcium and bone homeostasis and has recently been widely studied and focused on for its roles in various conditions and diseases, including skeletal (bone health) and non-skeletal health conditions such as infection, immunity, and cancer [1,2,3]. 

Vitamin D comes in the two forms of vitamin D2 and vitamin D3, both of which need to undergo chemical changes before they can be used by the body [2]. These changes occur in the liver and kidneys and convert vitamin D into the measurable substances 25-hydroxyvitamin D (25(OH)D) and 1,25-dihydroxyvitamin D (1,25(OH)2D) [1,2]. Because of the lower concentration of the circulating physiologically active form 1,25(OH)2D, the major circulating metabolite of vitamin D, 25(OH)D, is considered the best indicator of overall vitamin D status, deficiency, and insufficiency [1,2,4]. 

Various clinical guidelines available for estimating vitamin D status suggest different cutoffs for 25(OH)D level to define vitamin D sufficiency, insufficiency, and deficiency, i.e., <5 ng/mL, <10 ng/mL, <12 ng/mL, <20 ng/mL, or <30 ng/mL [3,5,6,7,8,9,10,11]. In Australia and New Zealand, vitamin D status is categorized as <5 ng/mL for severe deficiency, <12 ng/mL for moderate deficiency, <20 ng/mL for mild deficiency, and ≥20 ng/mL for sufficiency [10]. In Switzerland, vitamin D status is categorized as <10 ng/mL for severe deficiency, <20 ng/mL for deficiency, and ≥20 ng/mL for sufficiency [7]. In Korea, ≥20 ng/mL is regarded as the appropriate concentration for skeletal health by the Ministry of Health and Welfare and by the physicians’ guidelines for osteoporosis [12,13]. However, values <10 ng/mL for deficiency and <30 ng/mL for insufficiency (as determined by the Endocrine Society) are commonly used in local clinics and hospitals in Korea [9].

It has been reported that the serum 25(OH)D level can be influenced by sun exposure (altitude, season, sunscreen use); ethnicity; dietary intake; comorbidities; and the use of drugs and supplements [1,5,6,9,14,15]. Although a high prevalence of vitamin D deficiency has been reported, current information on vitamin D deficiency in the Korean population is limited. Furthermore, most previous studies about vitamin D status in the Korean population used data from earlier than 2017 or included a limited number of study subjects [9,14,16,17,18]. 

Because Green Cross Laboratories perform serum 25(OH)D tests as requested by local clinics and hospitals throughout Korea, we gathered recent information on test utilization and the seroprevalence of vitamin D deficiency in a large adult Korean population visiting local clinics and hospitals. The aim of this study was to investigate vitamin D status in the adult Korean population.

## 2. Materials and Methods

Serum 25(OH)D test results performed between 1 July 2017 and 31 December 2021 were retrospectively reviewed to investigate the 25(OH)D test results and the prevalence of vitamin D deficiency status. For 25(OH)D level determination, the Architect 25-OH vitamin D chemiluminescence microparticle immunoassay (Architect i2000SR; Abbott Laboratories, Chicago, IL, USA) was used. The measurement interval of the assay was 3.4–155.9 ng/mL. Analytical methods for 25(OH)D level in the laboratory were consistent during the study period. The accuracy of 25(OH)D tests was assured by participation in the proficiency testing program by the College of American Pathologists and the Korean Association of External Quality Assessment Service.

Data were excluded from the analysis when (1) patients had missing data for age or sex and (2) tests were repetitively performed in the same individual. Anonymized data were used for statistical analysis. Annual numbers of patients diagnosed with and managed for vitamin D deficiency in Korea were reviewed through a public database, the Healthcare Bigdata Hub by the Health Insurance Review & Assessment Service (HIRA), using the 10th revision, clinical modification of the International Statistical Classification of Diseases and Related Health Problems (ICD-10-CM) code E55 for vitamin D deficiency (available at: http://opendata.hira.or.kr/op/opc/olap3thDsInfo.do, accessed on 8 April 2022). Information on the numbers of the Korean population was obtained from the public database of the Korean Statistical Information Service by Statistics Korea (https://kosis.kr/eng/, accessed on 8 April 2022).

Because various clinical guidelines concerning vitamin D deficiency and insufficiency use different criteria for 25(OH)D level, we investigated the prevalence of vitamin D deficiency status according to various guidelines, including those from Australia/New Zealand (<5 ng/mL for severe deficiency [10]); the Federal Commission for Nutrition of Switzerland (<10 ng/mL for severe deficiency [7,8] = deficiency criteria commonly used in local clinics and hospitals in Korea [9]); the Institute of Medicine and Global Consensus for rickets (<12 ng/mL for deficiency [5,11]); and the Endocrine Society (<20 ng/mL for deficiency and <30 ng/mL for insufficiency [6,9]).

Numbers and percentages of each vitamin D status according to clinical guidelines set based on 25(OH)D level are presented. Vitamin D status and 25(OH)D level were investigated by age, sex, and month and year of test. For non-normally distributed quantitative variables (age and 25(OH)D level), non-parametric analyses (Mann–Whitney U tests) were used. Chi-square tests were used to compare the prevalence of each vitamin D status by year and age group. A value of *p* < 0.05 was considered statistically significant with the MedCalc statistical software version 19.1.5 (MedCalc Software bv, Ostend, Belgium). Ethical approval for this study was obtained from the Institutional Review Board of Green Cross Laboratories (GCL-2022-1012-01, 8 April 2022).

## 3. Results

**Figure 1:**
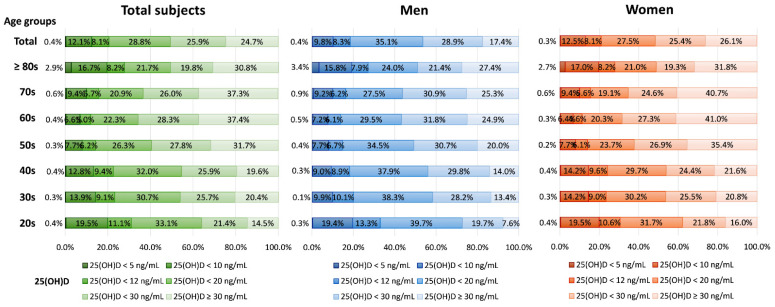
Prevalence of vitamin D deficiency status based on serum 25(OH)D level by sex and age.

During the study period, 180,289 subjects (29,658 men and 150,631 women) aged a median of 44.8 years (interquartile range (IQR) 33.2–56.0 years) were tested for serum 25(OH)D. The median 25(OH)D level of all subjects was 20.2 ng/mL (IQR, 13.0–29.8 ng/mL). The median 25(OH)D levels of men and women, respectively, were 19.0 ng/mL (IQR, 13.4–26.7 ng/mL) and 20.5 ng/mL (IQR, 12.9–30.5 ng/mL, *p* < 0.0001). The serum 25(OH)D level and number of subjects with each vitamin D status based on 25(OH)D level by sex and age are presented in Figure 1. The overall prevalence of vitamin D deficiency status based on 25(OH)D level was as follows: 0.4% for <5 ng/mL, 12.5% for <10 ng/mL, 20.6% for <12 ng/mL, 49.4% for <20 ng/mL, and <75.3% for <30 ng/mL. Women tested their 25(OH)D level more frequently than men, and the overall prevalence rate of women with 25(OH)D < 10 ng/mL was greater than that of men (12.8% in women vs. 10.2% in men, *p* < 0.0001), while that of women with 25(OH)D <30 ng/mL was lower than that of men (27.5% in women vs. 35.1% in men, *p* < 0.0001). Among the age groups, the prevalence of 25(OH)D < 30 ng/mL was higher in younger groups (20s–40s, 79.6–85.5%) than older ones (≥50 years, 62.6–69.2% *p* < 0.0001), and the median 25(OH)D level was lower in younger patients (20s–40s, 16.0–18.8 ng/mL) than older ones (≥50 years, 20.4–25.6 ng/mL, *p* < 0.0001).

**Figure 2:**
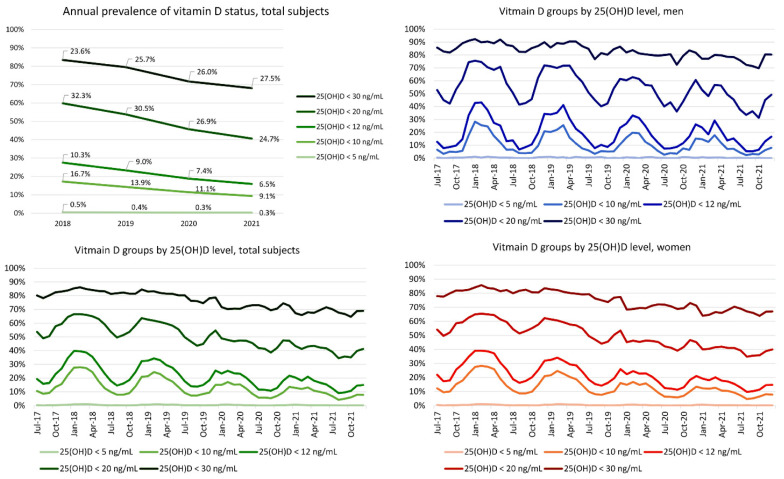
Median serum 25(OH)D level by sex, age, and test month. The total number of subjects with 25(OH)D level by month in the age groups of 70s and ≥80s was <100 for several months. Darker to brighter lines represent younger to older age groups. Men aged ≥80 years were not tested in some months.

**Figure 3:**
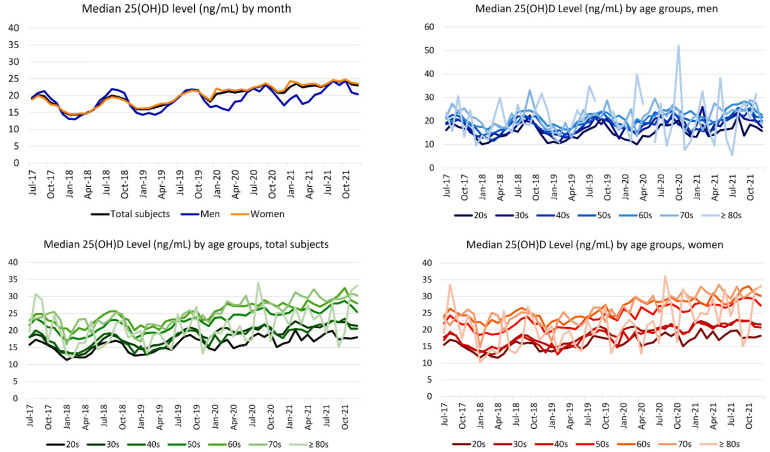
Prevalence of each vitamin D status based on 25(OH)D level by sex, age, and test month and year.

Because Korea is located at mid-latitude in the Northern Hemisphere, June to August is the summer season. The prevalence of subjects with 25(OH)D deficiency and median 25(OH)D level fluctuated by month (i.e., higher levels during summer and lower levels during winter). The median 25(OH)D level and the prevalence of each vitamin D status are presented in Figure 2 and Figure 3. The patterns of fluctuation of median 25(OH)D levels by month (changes by season) were similar between men and women. The median 25(OH)D levels slightly increased from 2017 to 2021.

The prevalence of each vitamin D status based on 25(OH)D levels decreased from 2018 to 2021 except when using the definition of deficiency of <30 ng/mL. For example, the prevalence of 25(OH)D < 20 ng/mL was 32.3% in 2018 and decreased to 24.7% in 2021. Meanwhile, the prevalence of 25(OH)D 20 ≤ 30 ng/mL was 23.6% in 2018 and increased to 27.5% in 2021. The prevalence of patients with a 25(OH)D level of 20 ≤ 30 ng/mL increased across the studied years. The seasonal fluctuation of the prevalence of each vitamin D status was similar in men and women.

According to the annual number of patients managed for vitamin D deficiency (ICD-10-CM code E55) reported in the Healthcare Bigdata Hub by the HIRA, the annual number of patients managed for vitamin D deficiency increased approximately twofold during the study period from 2018 (117,550 patients) to 2021 (247,077 patients). The number of patients managed for vitamin D deficiency by month was available from July 2017 to August 2021 (data for September to December 2021 were not available) and is presented in Supplementary Figure S1. Korean women were more commonly managed for vitamin D deficiency than were Korean men (821,426 women vs. 229,183 men) between July 2017 to August 2021. The annual percentage of patients managed for vitamin D deficiency between 2018 and 2020 was reviewed against the total number of Koreans according to Statistics Korea. Among sex and age groups, women in their 50s were managed most frequently for vitamin D deficiency and contributed to the annual increase in the prevalence of vitamin D deficiency.

## 4. Discussion

In this study, we investigated the recent information on the prevalence of vitamin D deficiency in an adult Korean population visiting local clinics and hospitals according to various clinical guidelines to define vitamin D deficiency. 

The overall prevalence of vitamin D deficiency status based on 25(OH)D level and the fluctuation by season were comparable to previous findings in Korean populations [9,14,16,17,19]. 

Previous guidelines and data concerning vitamin D status were inconsistent regarding the cutoffs for vitamin D deficiency. Although the cutoff <20 ng/mL is commonly used, the Scientific Advisory Committee on Nutrition, United Kingdom, 2016 and other studies use < 10 ng/mL to define deficiency [1,9,20]. The definitions of vitamin D statuses should be specified based on ranges of concentration, and clinical impacts of both skeletal and non-skeletal health in different populations should be further studied.

In this study, women tested their 25(OH)D level more frequently than did men, the overall prevalence of women with 25(OH)D < 10 ng/mL was higher than that of men, and that of women with 25(OH)D <30 ng/mL was lower than that of men. The number of patients managed for vitamin D deficiency (ICD-10-CM code E55) was skewed toward women according to the public database maintained by the HIRA. According to this public database, among sex and age groups, women in their 50s were managed most frequently for vitamin D deficiency and contributed most significantly to the annual increase in prevalence. However, in this study, among age groups, women in their 30s were tested the most frequently. Furthermore, the prevalence of vitamin D deficiency was higher in younger (20s–40s) participants than older ones (≥50 years), with corresponding findings that the median 25(OH)D level was lower in younger participants (20s–40s) than older ones (≥50 years). These findings are comparable to those of previous studies performed in Korean populations, such as among health examinees and in the 2008–2014 Korea National Health and Nutrition Examination Survey (KNHANES) [9,16]. However, in the study using KNHANES 2008–2014 data, an annual increase in vitamin D deficiency with an annual decrease in median 25(OH)D level was reported [16]. In the present study, the overall prevalence of vitamin D deficiency as defined by all criteria of 25(OH)D level decreased over time from 2018 to 2021 (except for the increase in the prevalence of patients with a level of 20 ≤ 30 ng/mL). The decrease in the prevalence of vitamin D deficiency contradicts the increased number of patients managed for vitamin D deficiency (ICD-10-CM code E55) from 2018 to 2021 according to the public database maintained by the HIRA. This difference might be due to the population characteristics of the subjects. This study mainly included adult Korean women visiting local clinics and hospitals, while patients with ICD-10-CM code E55 are all those managed for vitamin D deficiency at all types of medical facilities, including tertiary medical centers and university hospitals. Older patients and those with comorbidities or concerns about health behaviors, including visiting medical institutions, might have led to the differences in patient population characteristics [14]. In addition, a diagnosis of vitamin D deficiency (ICD-10CM code E55) was based on the physician’s report considering clinical findings, which is different from the population assessed in the present study, where we incorporated the serum 25(OH)D test result only. Furthermore, because the aim of this study was to investigate the prevalence of vitamin D deficiency based on 25(OH)D test results, repetitive test results were not included for analysis. The data from the HIRA contain repetitive patient information. Future studies are needed to clarify the significance of this difference in association with the clinical impact of vitamin D status [21].

Vitamin D3 is the naturally occurring form of vitamin D in humans during exposure to sunlight [1]. Vitamin D2 can be obtained through the consumption of food products that contain vitamin D2 naturally or by fortification [1]. In this study, serum 25(OH)D was measured using a chemiluminescence microparticle immunoassay that has been verified in the Korean population [22]. The performance evaluation and measurement uncertainty of the method used in the present study were verified using clinical specimens from the Korean population and SRM 972a (including materials with different levels of 25(OH)D2 and 25(OH)D3), revealing that it was adequate for uncertainty estimation in comparison with isotope dilution liquid chromatography tandem mass spectrometry (ID-LC-MS/MS) [22]. Although large population-based studies for 25(OH)D2 and 25(OH)D3 in a Korean population are limited, the KNHANES phase IX (2022–2024) includes the measurement of serum 25(OH)D2 and 25(OH)D3 levels using LC-MS/MS in a Korean population. 

The lack of clinical information associated with vitamin D deficiency or insufficiency, such as comorbidities and supplement use, is a limitation of the present study. However, because vitamin D deficiency is usually asymptomatic, the serum 25(OH)D level is commonly used to categorize vitamin D status [5,6]. The prevalence of vitamin D deficiency status based on 25(OH)D level according to various clinical guidelines investigated in this study might help to estimate the disease burden of vitamin D deficiency in adult Koreans. The large population analyzed over a recent long study period is a strength of the present study. The results of this study could be helpful for setting public health improvements for vitamin D status in the adult Korean population. Furthermore, this study might help to identify populations at greater risk of vitamin D deficiency who require further evaluation and management for nutritional support [5,6]. 

## 5. Conclusions

In conclusion, this study investigated vitamin D status in the adult Korean population visiting local clinics and hospitals. The 25(OH)D level was tested more frequently in women than men in this study. Different prevalence rates of vitamin D deficiency status were observed by sex, age, and month and year of testing. The overall prevalence of a low level of serum 25(OH)D was greater in men, younger age groups, and during winter. The overall prevalence of a low level of serum 25(OH)D decreased over time from 2018 to 2021 in the adult Korean population visiting local clinics and hospitals, which was different from the increase in the annual numbers of patients managed for vitamin D deficiency gleaned from a public database. The results of this study could be helpful for strengthening the understanding of vitamin D status in the Korean population.

## Data Availability

The datasets generated and analyzed during the current study are available from the corresponding authors on reasonable request.

## Supplementary Materials

The following supporting information can be downloaded at: https://www.mdpi.com/article/10.3390/nu14091978/s1, Figure S1: The number of patients managed for vitamin D deficiency from July 2017 to August 2021 (data from the Healthcare Bigdata Hub by the Health Insurance Review & Assessment Service, Korea) and the annual percent of patients managed for vitamin D deficiency from 2018 to 2020 (data from Statistics Korea).
