# Supplementary material for: Recent Information on Vitamin D Deficiency in an Adult Korean Population Visiting Local Clinics and Hospitals
Source: Nutrients. 2022 May 9;14(9):1978. doi: 10.3390/nu14091978 (PMC9101007; doi:10.3390/nu14091978)
Supplement: Supplementary file 1 [file nutrients-14-01978-s001.zip › nutrients-1701260-supplementary.pdf]

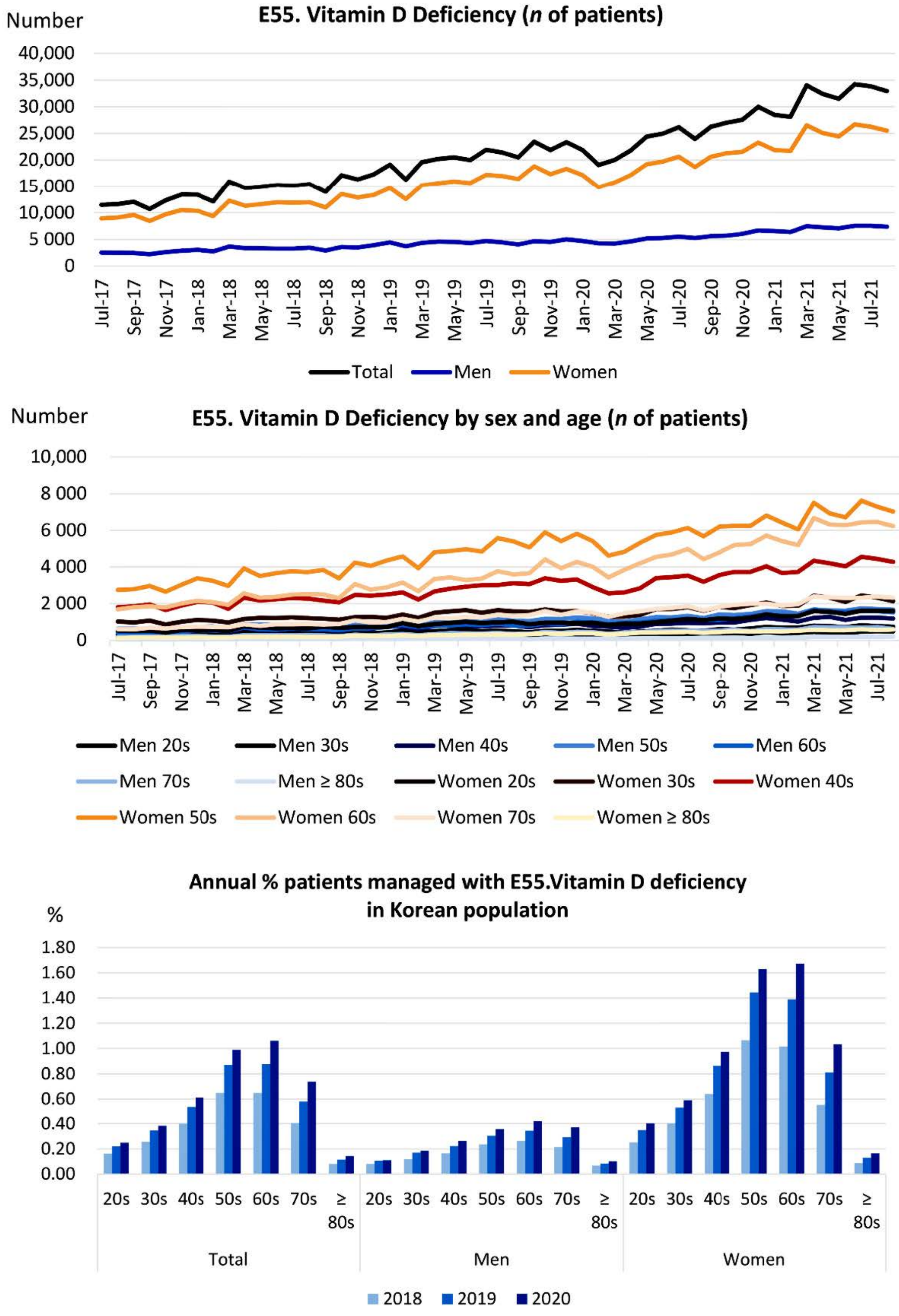


**Supplementary Figure S1.** The number of patients managed for vitamin D deficiency from July 2017 to August 2021 (data from the Healthcare Bigdata Hub by the Health Insurance Review & Assessment Service, Korea) and the annual percent patients managed for vitamin D deficiency from 2018–2020 (data from Statistics Korea). Top, data for all subjects. Middle, data by age group. Bottom, annual percentage of patients managed for vitamin D deficiency between 2018–2020. Darker to brighter lines represent younger to older age groups.
